# Congenital absence of the right iliac system with aneurysmal degeneration of a collateral lumbar artery: Diagnosis and treatment

**DOI:** 10.1016/j.jvscit.2025.101768

**Published:** 2025-03-04

**Authors:** John French, Zachary Schmittling, Robert Vorhies, Randolph Mullins, Scott Grant

**Affiliations:** a University of Missouri School of Medicine-Springfield Clinical Campus, Springfield, MO; b Cox Health System Department of Vascular Surgery and Department of Research and Innovation, Springfield, MO

**Keywords:** Absent right iliac artery, Angiography, Coil Embolization, Femoral-femoral bypass, Pseudoaneurysm

## Abstract

Congenital absences of the iliac arterial system are rare and often discovered incidentally. In these cases, treatment and operative strategy may require adjustment based on unique anatomy. In this report, we present a 67-year-old man who presented with a symptomatic large pseudoaneurysm of the lumbar artery secondary to absence of the iliac arterial system. Herein, we discuss treatment of this patient and review congenital arterial abnormalities.

The arterial anatomy of the human body varies from person-to-person. Anatomic differences exist, but the majority do not lead to any major issues. In rare cases, these variants can lead to aneurysmal formation, arterial thrombosis, and other complications.

In brief, angioblasts are the vascular precursor cells that organize into capillary plexuses in a process known as vasculogenesis. As the embryo grows, the primary capillary plexus must undergo reorganization by resorbing existing vessels and germinating new branches to support the expanding vascular network.^1^^,^^2^ If angiogenesis is disrupted during embryogenesis, abnormal arterial anatomy occurs. In this case, the agenesis of the common iliac artery occurred in the fourth week of fetal development, most likely involving an interruption in the umbilical arteries.^3^ This interruption possibly produced the absence of the dominant placental-aortic connection, which would have eventually branched off the aorta to become the common and internal iliac artery.^4^^,^^5^

A completely absent iliac arterial system is very rare. A 1977 study of 8000 patients who underwent pelvic angiography artery showed only six patients to have congenital absence of the iliac artery.^4^ A study by Koyama et al found incomplete formation of the external iliac artery to be extremely rare.^6^ These anatomic differences usually go undetected until an unrelated ailment or injury occurs, drawing attention to this abnormality via diagnostic imagining or operative findings.

Herein, we present a patient with absence of the right iliac system leading to formation of collateral vessels (the lumbar arteries) that, over time, developed aneurysmal degeneration. We will discuss his presentation and work-up. The surgical approach, both endovascular and open, are reviewed as well. Our patient provided written informed consent for the report of their case and imaging studies.

## Case report

**Fig 1:**
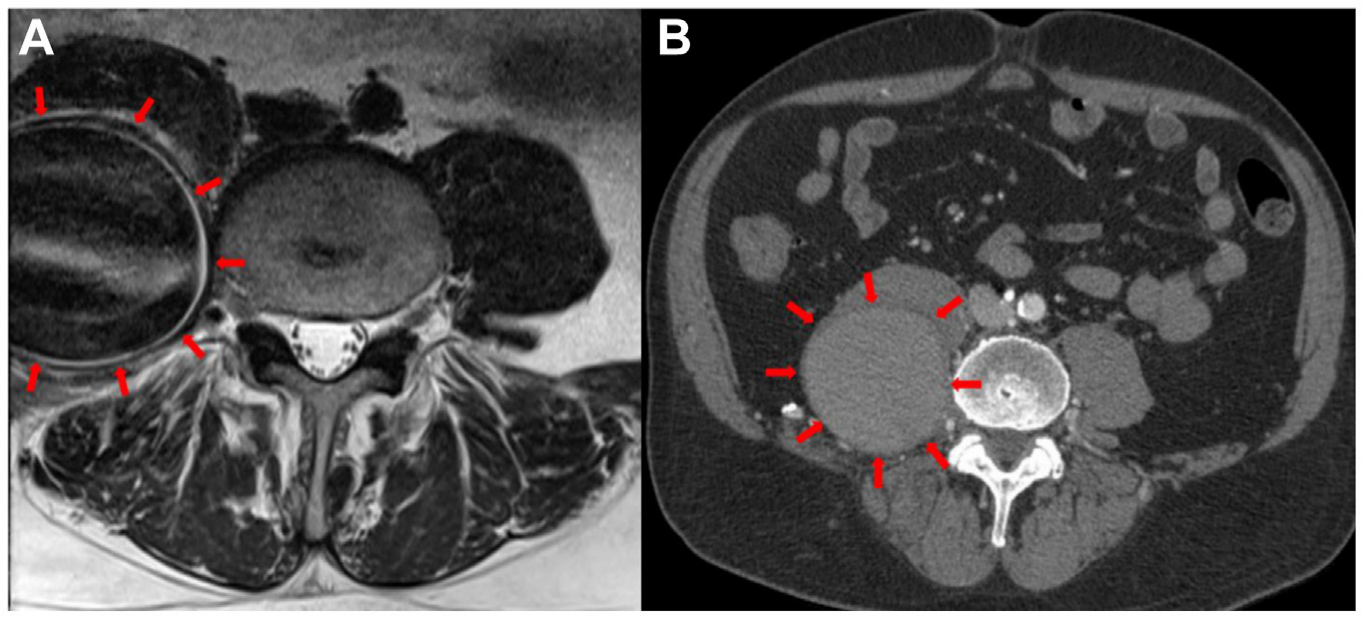
Pseudoaneurysm/aneurysm image acquisitions. **(A)** The left image is an axial T2 mangetic resonance acquisition. **(B)** The right image displays the aneurysm on computed tomography imaging. The dimension of the aneurysm was 6.2 × 6.5 × 8.4 cm in diameter. *Red arrows* illustrate the borders of the aneurysm in proximity to the right psoas muscle.

A 67-year-old man presented with lower, severe, chronic back pain radiating to the right lower extremity for 6 months. He was treated for standard lumbar pain without relief. He was seen by the neurosurgery service, who obtained magnetic resonance imaging. This revealed a large mass over the right psoas, which led to ordering of a computed tomography angiography (CTA). This showed a large aneurysm/pseudoaneurysm arising from a lumbar artery measuring 6.2 × 6.5 × 8.4 cm in diameter (Fig 1). Ankle-brachial indices (ABIs) were not available preoperatively. Left femoral and pedal pulses, but no right femoral pulse, could be obtained; Doppler displayed biphasic right pedal signals. Vascular surgery was consulted and proceeded with angiography.

**Fig 2:**
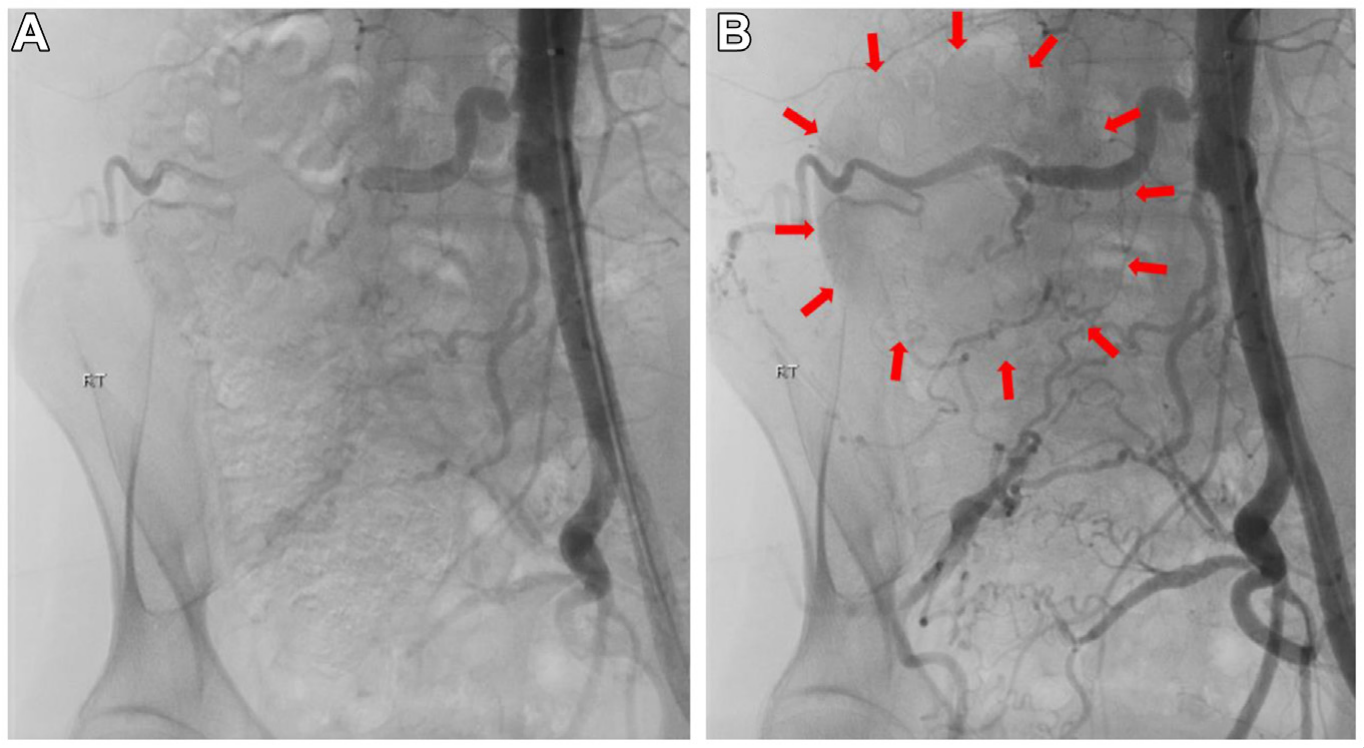
Angiography was performed, which showed absence of the iliac arterial system and a large pseudoaneurysm arising off one of the lumbar arteries. **(A)** Aneurysm prior to fluoroscopy. **(B)** Aneurysm when contrast is applied. The *red arrows* help to give better orientation to the borders of the aneurysmal formation.

**Fig 3:**
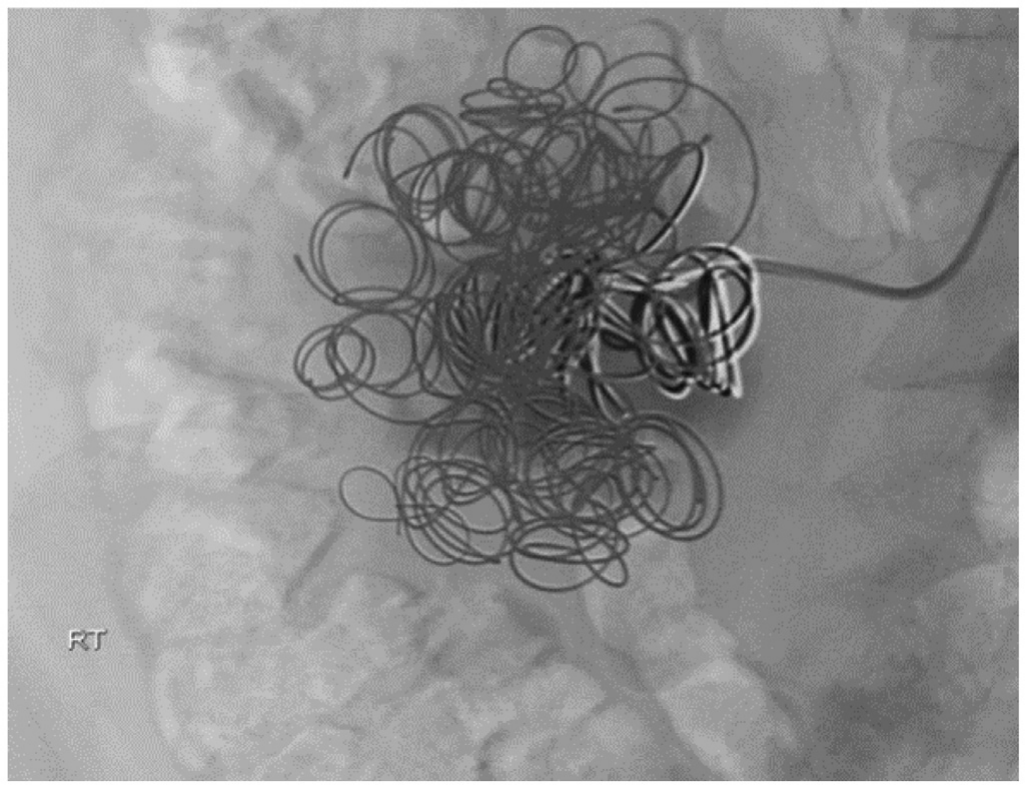
Angiogram of Nester coils that provided coil embolization of the aneurysm.

**Fig 4:**
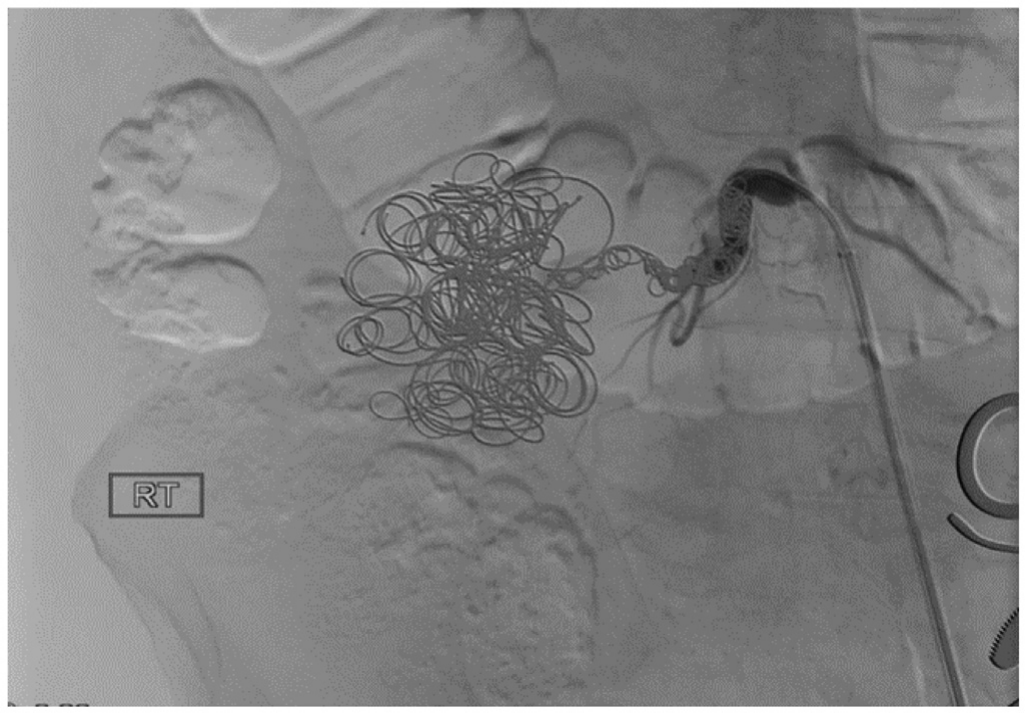
Angiogram illustration of complete embolization of aneurysm.

The patient had no history of significant surgery or trauma. He was a premature baby and, on further questioning, had a long history of claudication and weakness of the right lower extremity. Angiography was performed, which showed absence of the iliac arterial system and a large pseudoaneurysm arising off one of the lumbar arteries, which was the main runoff to the right lower extremity (Fig 2). Coil embolization of the aneurysm was performed with 20 Nestor coils ranging from 10 × 0.018 mm to 14 × 0.038 mm (Fig 3). Coil embolization of the feeding lumbar artery was not performed due to concerns of causing ischemic lower extremity. He followed up a week later without significant improvement. Lack of improvement was a result of the aneurysm still having flow, although significantly decreased, and increasing pain that began a day prior to undergoing endovascular coil catheterization. There also appeared to be a hematoma consistent with rupture that likely occurred at that time. Therefore, he was taken for coil embolization of the offending lumbar artery with planned femoral to femoral bypass grafting with 8 mm PTFE Dacron graft. A successful procedure was performed (Fig 4), and he was discharged home on postoperative day 2.

**Fig 5:**
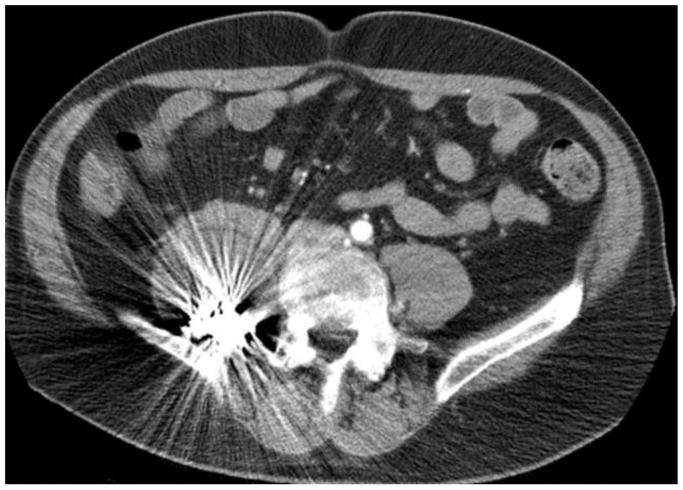
Computed tomography acquisition at 6-month follow-up illustrates a smaller aneurysmal sac as a result of coil embolization.

**Fig 6:**
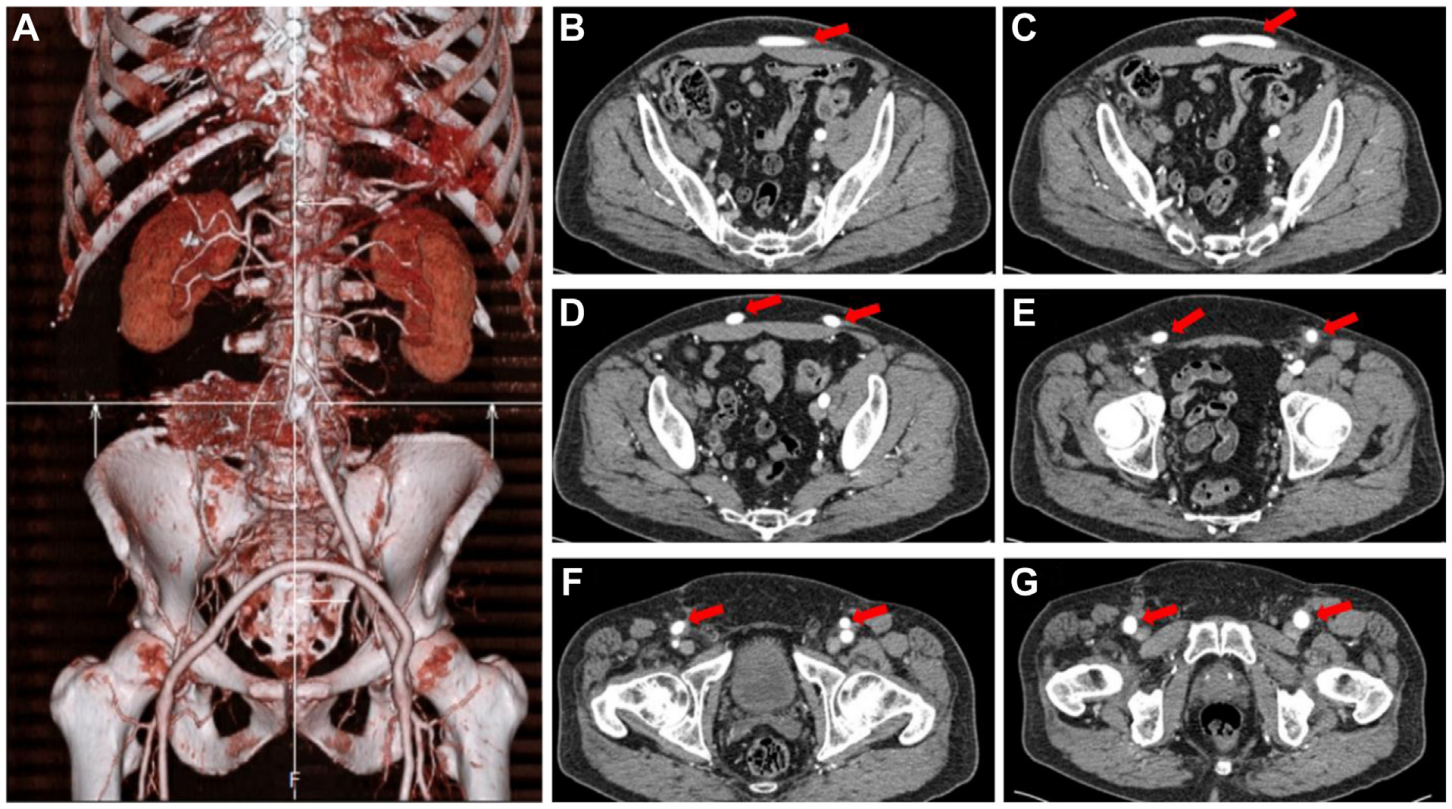
Bypass reconstruction and flow. **(A)** A three-dimensional computed tomography reconstruction of the patient’s bypass graft 6 months post operation. **(B-G)** Computed tomgraphy angiogram (CTA) of blood flow through the bypass graft. *Red arrows* correspond with the bypass graft until it merges into the femoral arteries bilaterally.

Immediately postoperatively, he experienced significant weakness and atrophy of the right lower extremity, which improved after 3 months of physical therapy. He reported complete resolution of his lumbar and right lower extremity pain. At 6-month follow-up, a CTA showed thrombosis of the aneurysm, with the sac measuring 4.5 × 3.0 cm in diameter (Fig 5). The CTA also provided information showing that the femoral-femoral bypass was still patent (Fig 6). ABIs were unfortunately not obtained due to other cardiovascular-related workup and treatment.

The patient was doing well 6 months after treatment of a large aneurysm arising from a congenital malformation. His treatment required coil embolization of the lumbar artery and femoral-femoral bypass.

## Discussion

During embryogenesis, the common iliac arteries result from the fifth lumbar arteries at the level of the fourth lumbar vertebra.^7^ When considering this developmental process, it is reasonable that a collateral vessel supplies blood to the lower extremity with absent common iliac vasculature. Collateral vasculature can include a persistent sciatic artery or a prominent lumbar artery.

We believe the mechanism of pseudoaneurysm formation was due to chronic high flow velocities through lumbar collateral arteries. Constant insult to lumbar vasculature would have caused damage that eventually resulted in an accumulation of blood between the tunica media and tunica adventitia of the artery. Over time, this continued to increase in size until it was large enough to cause the patient severe pain and rupture.

There was consideration for an aorta-unifemoral bypass rather than a femoral-femoral bypass, due to their presumably better patency rate long-term with an aortic-based reconstruction. This was not elected because the patient had previous heart valve replacement and coronary artery disease. Femoral-femoral bypass is used in selected patients when aortofemoral bypass is believed to be inappropriate because of high operative risk or predominantly unilateral iliac artery occlusive disease, which in this case was the absence of a common iliac artery.^8^

Congenital absences of the iliac system are rare, often not problematic for the patient, and usually discovered during testing for related conditions. In fact, Doita et al found only 12 cases of congenital common iliac artery absence reported from 1964 to 2021. In the 12 cases they described, nearly all the cases of congenital absence of the common iliac arteries were diagnosed incidentally.^9^ Similarly to our case, individuals tended to present with claudication and/or lumbar pain. A 2022 case study showed a patient with congenital absence of left common and external iliac arteries. This patient was treated conservatively over the course of 6 years, both ABIs and toe-brachial indices remained stable, and no ischemic symptoms were noted.^10^ Doita et al recommended that careful preoperative assessment is required before performing surgery or a catheter-based intervention and to consider bypass procedures if ischemia symptoms worsen.^9^ Our patient was observed to have increasing ischemic pain due to his condition, which is why bypass was eventually considered as a treatment option.

When an iliofemoral anomaly is observed, it is essential to inform the patient and also to confirm whether other organ anomalies are present. Ischemic symptoms are likely to appear if the collateral circulation is damaged; thus, we approached this symptomatic patient with a hybrid type procedure. It was felt that an open intra-abdominal reconstruction would be difficult due to location of the aneurysm and likely friability of the surrounding tissues. The hybrid approach led to a good result, with thrombosis of the aneurysm and resolution of the patient’s symptoms.

## Conclusions

This rare case shows the importance of imaging prior to intervention for lumbar pain. In addition, it highlights the combined (endovascular and open) approach for the difficult vascular patient.

## Funding

None.

## Disclosures

None.

## References

[1] De Val S. (2011). Key transcriptional regulators of early vascular development. Arterioscler Thromb Vasc Biol.

[2] Iruela-Arispe M.L., Davis G.E. (2009). Cellular and molecular mechanisms of vascular lumen formation. Dev Cell.

[3] Mirilas P., Skandalakis J.E. (2010). Surgical anatomy of the retroperitoneal spaces, part III: retroperitoneal blood vessels and lymphatics. Am Surg.

[4] Greeb J. (1977). Congenital anomalies of the iliofemoral artery. J Vasc Surg.

[5] Sato Y. (2013). Dorsal aorta formation: separate origins, lateral-to-medial migration, and remodeling. Dev Growth Differ.

[6] Koyama T. (2003). Congenital anomaly of the external iliac artery: a case report. J Vasc Surg.

[7] Senior H.D. (1919). The development of the arteries of the human lower extremities. Am J Anat.

[8] Schneider J.R., Besso S.R., Walsh D.B., Zwolak R.M., Cronenwett J.L. (1994). Femorofemoral versus aortobifemoral bypass: outcome and hemodynamic results. J Vasc Surg.

[9] Doita T., Yamakura T., Yamasumi T., Nakamura T. (2021). Congenital absence of left common and external iliac arteries. J Vasc Surg Cases Innov Tech.

[10] Harb Z., Williams S., Rutter P. (2006). Bilateral congenital absence of internal iliac arteries, prominent lumbar arteries, and a ruptured mycotic aneurysm of the abdominal aorta. Ann R Coll Surg Engl.

